# Visual Speech Perception in Foveal and Extrafoveal Vision: Further Implications for Divisions in Hemispheric Projections

**DOI:** 10.1371/journal.pone.0098273

**Published:** 2014-07-17

**Authors:** Timothy R. Jordan, Mercedes Sheen, Lily Abedipour, Kevin B. Paterson

**Affiliations:** 1 Department of Psychology, Zayed University, Dubai, UAE; 2 School of Psychology, University of Leicester, Leicester, United Kingdom; Cardiff University, United Kingdom

## Abstract

When observing a talking face, it has often been argued that visual speech to the left and right of fixation may produce differences in performance due to divided projections to the two cerebral hemispheres. However, while it seems likely that such a division in hemispheric projections exists for areas away from fixation, the nature and existence of a functional division in visual speech perception at the foveal midline remains to be determined. We investigated this issue by presenting visual speech in matched hemiface displays to the left and right of a central fixation point, either exactly abutting the foveal midline or else located away from the midline in extrafoveal vision. The location of displays relative to the foveal midline was controlled precisely using an automated, gaze-contingent eye-tracking procedure. Visual speech perception showed a clear right hemifield advantage when presented in extrafoveal locations but no hemifield advantage (left or right) when presented abutting the foveal midline. Thus, while visual speech observed in extrafoveal vision appears to benefit from unilateral projections to left-hemisphere processes, no evidence was obtained to indicate that a functional division exists when visual speech is observed around the point of fixation. Implications of these findings for understanding visual speech perception and the nature of functional divisions in hemispheric projection are discussed.

## Introduction

The facial movements that accompany speech production (*visual speech*) are a powerful component of speech perception [1]–[11]. In particular, seeing the articulating face of a talker can improve auditory speech intelligibility substantially in quiet and noisy environments and, in the McGurk effect [6], can alter the perceived identity of speech sounds. However, although these effects are well-established, the processes underlying perception of visual speech have yet to be fully revealed.

An important aspect of visual speech perception that has been largely overlooked is the manner in which information from a talking face projects to the cerebral hemispheres of the observer. In particular, a fundamental determinant of hemispheric processing for any visual input is the anatomical arrangement of the human visual system which causes areas in each visual hemifield to project unilaterally to the contralateral hemisphere. Consequently, visual speech encountered in locations to the left of fixation may project only to the right hemisphere (RH) and visual speech encountered in locations to the right of fixation may project only to the left hemisphere (LH), and this division is likely to have important consequences for how visual speech is processed. Indeed, several studies have shown that, when a talking face is observed, although visual speech ultimately produces activation in both hemispheres, activation is more extensive in the LH, in areas known to be involved in auditory speech perception, and this is consistent with the dominant role of the LH in processing language ([12]–[15], see also [16]). Thus, although the RH is implicated in many aspects of facial processing (for a review, see [17]), processes located in left cortex seem to dominate visual speech perception. As a result, when visual speech is observed, perception of visual speech is likely to benefit when it is encountered in locations that project directly to the LH. Indeed, empirical support for this benefit comes from a study by Jordan and Thomas [16] in which talking faces were presented in either the left or right hemifield, in locations 2° away from the point of fixation. The findings showed that identification of visual speech was superior for faces presented in the right hemifield, suggesting that right hemifield projections to LH processes play an important functional role in visual speech perception.

However, the nature and influence of hemispheric projections for visual speech perception in areas closer to fixation remain to be determined. Of particular interest is that it is well established that visual information presented to the left and right sides of each retina outside the fovea projects to each contralateral hemisphere (for reviews, see [18]–[21]) but the associated view that this division in hemispheric projections does not extend up to the point of fixation has attracted some opposition.

On the one hand, a considerable body of evidence indicates that the fovea contains an intermingling of ganglion cells around the foveal midline that project contralaterally and ipsilaterally in an area typically regarded as extending 1–2° each side of the midline, so that information falling within this area projects directly to both hemispheres (see [18], [21]–[30]). (This is why many researchers using lateralised displays to investigate hemispheric processing present stimuli outside this central area of bilateral projection. For discussions, see [16], [19]–[21], [31]–[33]). Thus, according to this view, visual speech falling within an area of foveal vision close to fixation is likely to experience the same pattern of hemispheric projections either side of the midline. In recent years, however, some researchers (e.g., 34) have revived the alternative suggestion that foveal vision is divided precisely at the midline so that visual information each side of fixation projects only to the contralateral hemisphere (for reviews, see [26], [35]). Most importantly, according to the view adopted by this *split fovea theory* (hereafter SFT), the division in hemispheric projections between the two hemifields is so absolute and precise at the vertical midline that even if a talking face were observed at the point of fixation, *all* visual speech to the left of the midline would project *only* to the RH and *all* visual speech to the right of the midline would project *only* to the LH. Historically, the notion that a precise split in hemispheric projections exists at the point of fixation was considered, investigated, and rejected some years ago by Mishkin and Forgays [36] when considering written word recognition. Nevertheless, if the SFT view is correct, visual speech encountered in the left and right hemifields would project entirely to different (contralateral) hemispheres even when encountered close to the point of fixation.

These two views provide contrasting predictions about the perception of visual speech lying to the left and right of fixation. From previous evidence of LH dominance and functional hemispheric projections for visual speech stimuli [12]–[16], [37]), visual speech shows evidence of a right hemifield advantage. However, if this functional division in hemispheric projections does not extend to the midline, only visual speech presented sufficiently far from fixation (e.g., in extrafoveal locations) should project unilaterally to each contralateral hemisphere and so produce a right hemifield advantage, whereas visual speech presented close to fixation (i.e., in foveal locations) should produce similar levels of performance in each hemifield. In contrast, if a functional division in contralateral projections extends right up to the midline (as SFT proposes), a right hemifield advantage should be observed even for stimuli presented close to the point of fixation.

Assessing perception of visual speech to the left and right of fixation is complicated by the absence of natural perfect symmetry between left and right hemifaces [16], [38]–[45]. Indeed, research measuring facial movements has revealed evidence of hemiface asymmetries in talkers’ articulations where the left side of the mouth (we refer to locations on the face from the observer’s perspective; e.g., left  =  left from the observer’s point of view) opens sooner and wider during speaking, probably due to LH control over speech production [41], [46]–[49]. Moreover, there is also evidence to suggest that this asymmetry in speech production may affect visual speech perception [16], [49]–[52] and these findings clearly have important implications for studying perception of visual speech in each hemifield. In particular, because visual acuity decreases as retinal eccentricity increases, if a normally articulating face were presented to the right of fixation, the left hemiface would fall in an area of visual acuity that was higher than for the right hemiface. Conversely, if the same face were presented to the left of fixation, the right hemiface would now have an acuity advantage over the left hemiface. Without appropriate stimulus control, therefore, asymmetry in hemiface information may inspire spurious effects on the perception of visual speech to the left and right of fixation because of mismatches in the basic visibility of this information and not because of differences in hemispheric processing.

**Figure 1:**
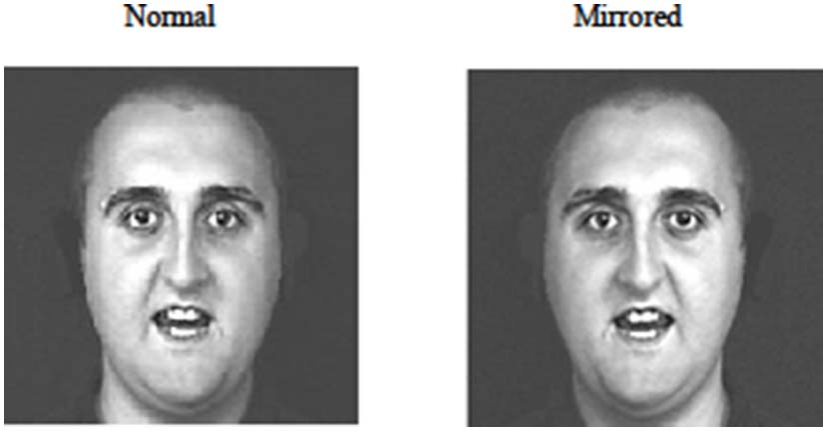
The facial displays used in the experiment. Normal displays were as recorded and mirrored displays were rotated 180° in the horizontal plane.

In light of these issues, the present study was conducted to reveal the functional division in hemispheric projections that exists for visual speech perception by using lateralised displays in which a talking face was presented to the left and right of the foveal midline either adjacent to fixation (in foveal locations) or further from fixation (in extrafoveal locations). Each facial image was presented as it was recorded (normal) and as a mirror image (mirrored; see Figure 1) so that the relative position and retinal eccentricity of each hemiface were matched across the two hemifields. In addition, the locations of all displays relative to the foveal midline were determined precisely by using an automated, gaze-contingent eye-tracking technique. Accordingly, by using these procedures, the experiment provided a highly accurate means of assessing hemifield asymmetries in visual speech perception at different eccentricities from fixation.

## Method

### Ethics Statement

This research was conducted in accordance with the ethical approval of the School of Psychology Ethics Committee at the University of Leicester, and in accordance with the ethical guidelines of the British Psychological Society. The individual used to produce the visual speech stimuli and whose image appears in this article (Figure 1) has given written informed consent (as outlined in the PLOS consent form) to publish his details and for the image to be included. All participants in the experiment gave informed consent in writing.

### Participants

Twenty native speakers of British English, aged 18–25, participated in the experiment. All participants were English, had at least normal or corrected to normal visual acuity, determined by a Bailey-Lovie Eye Chart, and were right-handed, determined by a revised Annett Handedness Questionnaire [53].

### Stimuli and Apparatus

Stimuli were created by recording the face of a 25-year old male native-English speaker while he fixated an HD video camera. Frame-by-frame analysis of the recorded footage confirmed that the speaker exhibited the faster and larger mouth movement on the left side of the face typical of speakers [16], [41], [46]–[49]. To remove confounding influences of head and facial hair, the speaker had closely cropped head hair and was clean shaven. The speaker’s face was fully illuminated and recorded with head stationary against a uniform dark-grey background with only the face and upper neck visible. Camera and lighting were configured so that the recorded face was reproduced life-sized and in natural colour on a colour display monitor which was used to monitor recordings and to display facial images in the experiment. Luminance was equated across the left and right hemifaces, as determined by a Minolta photometer at the speaker’s face and by a Cambridge Research Systems ColorCAL at the monitor.

Recordings were made of the speaker saying each of six consonant-vowel utterances in an English accent. Each utterance comprised a consonant,/b/,/g/, or/v/, followed by one of two vowels,/a/or/i/. Each articulation began and ended with the face in neutral repose (mouth closed) and each syllable was spoken naturally with no artificial emphasis on articulation. These recordings were then imported into post-production editing software and a single clip of each syllable (/ba/,/bi/,/ga/,/gi/,/va/,/vi/) was then used to produce the experimental stimuli.

Each clip was shown in two different forms: normal, in which each facial image was presented as it had been recorded, and mirrored, in which each facial image was rotated 180° in the horizontal plane so that the relative positions of the left and right hemifaces were reversed (see Figure 1). In addition, the size of each normal and mirrored facial stimulus was adjusted for foveal and extrafoveal locations to avoid confounding differences in visibility on overall levels of performance [54]. Specifically, foveal stimuli subtended 1° wide and were presented to either the left or right of a central fixation point so that the medial (inner) edge of each facial image abutted either the left or right side of the fixation location. Extrafoveal stimuli subtended 2° wide and the medial edge of each facial image was 2° from either the left or right side of the fixation location. Preliminary testing had established that these sizes and eccentricities produced similar levels of overall performance for foveal and extrafoveal displays and helped ensure that stimuli were shown entirely in either foveal or extrafoveal locations.

For each display, the face remained static until the onset of the articulation which occurred 2 seconds after the onset of the display. Each display lasted 6 seconds in total and was followed by a 6 second blank, during which participants made their response using a mouse to select from an array of twelve possible responses presented on a screen: “ba”, “bi”, “bga”, “bgi”, “da”, “di”, “ga”, “gi”, “tha”, “thi”, “va”, “vi”. Pre-testing had established that these responses constituted more than 97% of participants’ perceptions of all stimuli used in the experiment.

Precise control of retinal location is crucial for hemifield research [19]–[21], [31], [33], [55] but this precaution has regularly been overlooked by studies supporting SFT (for evidence of this oversight and its implications, see [19]–[21], [31], [55]–[60]). Accordingly, in the present study, each participant’s fixation location was monitored using a Skalar IRIS eye-tracking system (Cambridge Research Systems) linked to the ADC input of a Cambridge Research Systems VSG2/5 card. The eye tracker was clamped to each participant’s head, which in turn was clamped in a head brace throughout the experiment to prevent head movements. This arrangement allowed accurate and consistent measurement of fixation location in the experiment (for further details, see [16], [32], [61], [62]). The output of the tracker was recorded through the ADC input of the VSG2/5 card, which also controlled the visual display.

### Design

Stimuli were shown in two sessions of 192 trials, each session corresponding to 8 presentations of the 6 speech stimuli shown normal and mirrored at each of the 2 eccentricities (foveal, extrafoveal). Within each session, all stimuli were displayed in either the left or right hemifield and participants fixated a fixation point located at either the left or right side of the presentation screen. The left-sided fixation point was fixated for right hemifield presentations and the right-sided fixation point was fixated for left hemifield presentations. To avoid disruption, the same fixation point was fixated throughout each session. The order of each hemifield session was counterbalanced across participants. In each session, all 192 displays were shown in a different random order. Each participant used only their left or right hand to make responses (via the mouse) and the allocation of response hand was counterbalanced across participants for each order of hemifield session.

### Procedure

Each participant was seated in a sound-attenuated room 1 m in front of the display screen, with their head level with the screen. Each session began by calibrating the eye tracker. For each session, participants fixated a fixation point located at either the left or right side of the presentation screen. At the start of each trial, fixation location was monitored until fixation of the fixation point occurred for 250 ms. The clip for the trial was then played while fixation of the fixation point continued to be monitored. If accurate fixation was lost during stimulus presentation, the display screen immediately went blank and the clip was presented later in the experiment. Approximately 8% of displays required repeat presentation. Participants were required to identify the syllable articulated on each trial by selecting a response from the options presented on the response screen after each stimulus had been shown. When questioned at the end of the experiment, all participants indicated that they had not been restricted in their responses by the options provided.

## Results

**Figure 2:**
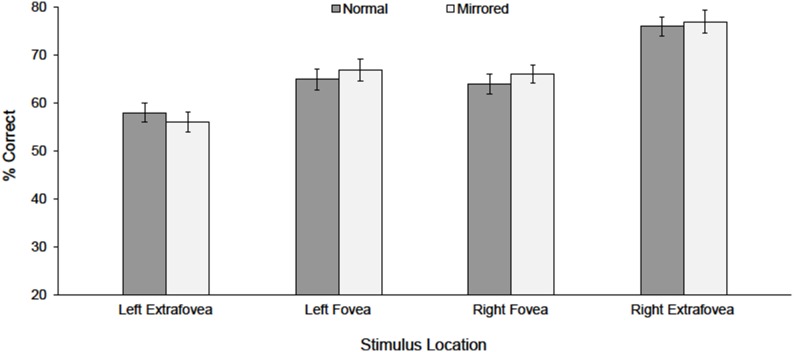
Mean percentages (% Correct) and standard errors for stimuli correctly identified in each location.

Mean identification accuracy for each presentation location is shown in Figure 2. Overall levels of performance were closely matched for foveal (66%) and extrafoveal (67%) stimuli, indicating that the size manipulations used in the experiment successfully matched overall stimulus visibility across foveal and extrafoveal locations. The data were analyzed using a 4-way within-participants ANOVA with variables hemifield (left, right), eccentricity (foveal, extrafoveal), display type (normal, mirrored), and speech stimulus (/ba/,/bi/,/ga/,/gi/,/va/,/vi/). The ANOVA showed a main effect of hemifield (left hemifield 62%, right hemifield 71%), *F*(1, 19) = 60.30, *p*<.0001, η_p_^2^ = .76, and an interaction between hemifield and eccentricity, *F*(1, 19) = 70.55, *p*<.0001, η_p_^2^ = .79. Tukey tests showed that this interaction was due to a substantial and highly significant right hemifield advantage for extrafoveal stimuli (left hemifield 57%, right hemifield 77%; *p*<0.0001) and no significant effect of hemifield for foveal stimuli (left hemifield 66%, right hemifield 65%, *p*>.50). Indeed, for foveal stimuli, performance was marginally higher for left hemifield stimuli than for right, and this underscores the lack of evidence of a right hemifield advantage for stimuli presented at the foveal midline. Finally, a main effect of speech stimulus was also found, *F*(5, 95) = 320.29, *p*<.0001, η_p_^2^ = .94, and Tukey tests showed that/ga/and/gi/produced fewer correct responses than any other speech stimulus (*p*s<0.0001). No other main effects or interactions were significant.

## Discussion

The purpose of this study was to investigate the functional division that exists in hemispheric projections for visual speech perception by using a lateralized viewing procedure in which a talking face was presented to the left or right of a fixation point in either foveal or extrafoveal locations. Of particular interest were the implications of two contrasting views concerning the projection of information around the foveal midline. On the one hand, a considerable body of evidence indicates that visual information around the foveal midline projects simultaneously to both hemispheres and so visual speech falling within this area should undergo the same pattern of hemispheric projections either side of fixation. On the other hand, an alternative view (SFT) proposes that foveal vision is divided precisely at the midline and so all visual information each side of fixation projects only to the contralateral hemisphere. Most importantly, according to SFT, the division in hemispheric projections that exists at the confluence of the two hemifields is so absolute and precise that *all* visual speech to the left of the midline will project *only* to the RH and *all* visual speech to the right of the midline will project *only* to the LH, with clear implications for hemifield processing.

The findings of this study revealed a pattern of visual speech perception to the left and right of fixation that clearly differed between foveal and extrafoveal locations. In particular, whereas visual speech in extrafoveal locations produced a strong right hemifield advantage, visual speech in foveal locations produced levels of performance that showed no evidence of any hemifield advantage and that were essentially identical on each side of fixation. Moreover, these findings were obtained for both normal and mirrored facial displays, using precisely controlled retinal locations, and in an experimental paradigm that was clearly well-suited to revealing differences in hemifield performance. Consequently, it seems reasonable to consider that the distinction between the effects of extrafoveal and foveal displays that was observed was not confounded by the retinal eccentricity of each hemiface, or by imprecise control of retinal locations, or by the use of an insensitive experimental technique.

The findings obtained with extrafoveal locations indicate that important functional unilateral projections to different, contralateral hemispheres exist for perception of visual speech outside foveal vision, and this is consistent with the findings of Jordan and Thomas [16] who also found a right hemifield advantage for lateralized displays of visual speech presented 2° from fixation. Consequently, although visual speech may produce activation in both hemispheres, the findings of the present study underscore the view that dominant processes of visual speech perception are located in the LH, and this is in accord with the role of the LH for processing language. However, our findings for foveal displays of visual speech stimuli (which, in our experiment, extended up to 1° either side of fixation) provide no evidence for the SFT view that each fovea is split precisely at the vertical midline and, as a consequence, no evidence that the functional division in hemispheric processing observed for extrafoveal locations extends to the foveal midline.

Moreover, while a precise split at the foveal midline is also unsupported by any clear anatomical evidence (see [21], [56]), it is interesting to note that the findings obtained in the present study suggest that even if an anatomical split in foveal processing existed along the lines proposed by SFT, this split has no functional relevance for visual speech perception. In particular, advocates of SFT argue that, because interhemispheric transmission is costly, the anatomical split in each fovea proposed by SFT means that projection to the nondominant hemisphere incurs processing costs even in foveal vision [63]–[66]; see also [34]. The findings we report for visual speech stimuli within foveal vision evidently do not support this view and suggest instead that, if human foveae were precisely split anatomically at the midline, the transmission of information between the two hemispheres is sufficiently rapid to obviate a functional role for this anatomical divide. Indeed, as Dehaene, Cohen, Sigman, and Vinckier [67] have pointed out, callosal projections beyond V1 may have the structure necessary to ensure the continuity of receptive fields across the foveal midline and to allow convergence on common visual representations, which may, therefore, remove the functional impact of any initial foveal split, even in the unlikely scenario that one actually existed (see also [68]).

The absence of support for SFT in the present study using visual speech stimuli resonates with the findings of previous studies in which the viability of SFT has been drawn into doubt using other types of visual stimuli (notably written words and nonwords; [21], [31], [56]–[60], [68]–[72]) and measures of both accuracy and reaction time. Moreover, the finding that effects of hemispheric asymmetry on visual speech perception were apparent for extrafoveal presentations while being entirely absent for foveal presentations adds important new support to the view that a sizable area of overlap exists around the point of fixation within which information projects bilaterally (to both hemispheres). Indeed, several previous studies have suggested that the area of functional bilateral projections around the foveal midline may extend up to 1° either side of fixation ([18], [24], for reviews, see [21], [31]), and this is consistent with the horizontal extent of the foveal visual speech stimuli used in our study.

The shift in hemifield performance observed between extrafoveal and foveal locations has implications for understanding how visual speech is processed in different parts of the visual field in everyday life. Most obviously, when encountering talking faces in extrafoveal locations, visual speech is likely to be perceived better in the right hemifield than in the left, but the picture now appears to be more complex than this. In particular, while our study used complete faces presented either side of fixation, the findings obtained for foveal displays suggest that, when fixating a talking face directly, a substantial area of the face around the foveal midline will project to both hemispheres. For example, at a viewing distance of 1 m, a talking face 140 mm wide (about typical for a human adult) will subtend a horizontal visual angle of approximately 8°. Consequently, when fixating centrally on this face, a substantial central strip is likely to project bilaterally (to both hemispheres) while areas to the left and right of this strip will project separately to different, contralateral hemispheres. Moreover, in line with the findings of the present study, this three-way segregation would produce bilateral projections for information around the midline that would include beneficial projections to the LH, but the greatest area of LH projections would be to the right of the central area of bilateral projection, where visual speech perception is likely to benefit. However, the precise contribution to visual speech perception made by this pattern of hemispheric projections will also depend on the distance at which a face is viewed and, indeed, its physical size. For example, useful visual speech can be obtained at considerable viewing distances, at least up to 30 m [73]; see also [74], and so the pattern of hemispheric projection of visual speech from a fixated face may vary considerably with viewing distance, and even be completely bilateral at some distances (over 4 m for an area of bilateral projection 2° wide at the midline). Thus, the findings of this study highlight a complex relationship between natural viewing conditions and hemispheric projections that must be accounted for when addressing the processes underlying visual speech perception and, indeed, that should be controlled when presenting visual speech stimuli in experiments, where vagaries in viewing distance and/or image size may substantially affect the results (see [73], [74]).

In sum, by precisely controlling the form and location of lateralized displays of visual speech stimuli, the present study has shown that functional unilateral projections to different, contralateral hemispheres exist outside foveal vision but that no such division is present at the foveal midline. Consequently, when considering the processes involved in visual speech perception from retina to cortex, it seems reasonable to conclude that while a functional division in hemispheric projections exists for visual speech in locations away from an observer’s point of fixation, this division does not extend to the point of fixation, and shows no influence on visual speech perception within foveal vision.
